# Preduodenal Portal Vein With Midgut Non-rotation Causing Duodenal Obstruction in a Neonate: A Case Report

**DOI:** 10.7759/cureus.106898

**Published:** 2026-04-12

**Authors:** Afsheen Umer, Quds Alani, Rawan Alhalabi, Rajesh Kewlani, Shyam Mohan, Gursev Sandlas, Sridhar Ramaiah

**Affiliations:** 1 Pediatrics, American Hospital Dubai, Dubai, ARE; 2 Surgery, American Hospital Dubai, Dubai, ARE; 3 Pediatric Radiology, American Hospital Dubai, Dubai, ARE; 4 Pediatric Surgery, American Hospital Dubai, Dubai, ARE; 5 Neonatology, American Hospital Dubai, Dubai, ARE

**Keywords:** congenital intestinal malrotation, congenital vascular anomaly, duodenal obstruction, neonatal bilious vomiting, preduodenal portal vein

## Abstract

Preduodenal portal vein (PDPV) is a rare congenital vascular anomaly that may cause duodenal obstruction, either in isolation or in association with other gastrointestinal malformations such as intestinal malrotation. We report a neonate who presented on the seventh day of life with bilious vomiting. Imaging studies suggested proximal duodenal obstruction due to extrinsic compression, with findings consistent with a preduodenal portal vein and associated midgut non-rotation. The diagnosis was confirmed intraoperatively, and the infant was successfully managed with duodenoduodenostomy and Ladd’s procedure.

Although uncommon, PDPV should be considered in neonates presenting with bilious vomiting and proximal intestinal obstruction. Early recognition is essential for guiding appropriate surgical planning and preventing potentially catastrophic intraoperative vascular injury.

## Introduction

Preduodenal portal vein (PDPV) is a rare congenital vascular anomaly in which the portal vein courses anterior to the duodenum rather than posteriorly, as observed in normal anatomy. This anomaly was first described by Knight in 1921 in his report titled “an anomalous portal vein with its surgical dangers” [1]. PDPV arises due to aberrant embryogenesis of the vitelline venous system. Normally, the portal vein develops through selective regression and anastomosis of the paired vitelline veins posterior to the duodenum. Persistence of the ventral venous channel with involution of the dorsal channel results in a preduodenal course of the portal vein [1,2].

PDPV is frequently associated with congenital anomalies involving laterality and foregut development [3]. Reported associations include situs inversus, heterotaxy and polysplenia syndromes, intestinal malrotation or non-rotation, annular pancreas, biliary atresia, duodenal atresia or stenosis, and complex congenital cardiac malformations [3-6].

Clinically, PDPV may remain asymptomatic and be detected incidentally during imaging or abdominal surgery. However, in the neonatal period, it may present as duodenal obstruction, either secondary to intrinsic anomalies such as duodenal atresia or due to extrinsic compression by an anomalous vein [6,7]. Recognition of this anomaly is particularly important intraoperatively, as inadvertent injury during procedures such as duodenoduodenostomy or Ladd’s procedure can result in catastrophic hemorrhage and compromise of the portal venous system. Given its rarity and frequent coexistence with other congenital anomalies, reporting cases of PDPV contributes to improved understanding of its embryological origin, clinical spectrum, and surgical implications.

## Case presentation

A term baby boy, born at 37 weeks + three days of gestation via spontaneous vaginal delivery, with a birth weight of 3.0 kg, Apgar scores of 9 and 10, required no resuscitation. He received vitamin K at birth and established breastfeeding early. The initial postnatal course was uneventful, apart from mild hypoglycemia that resolved with feeds, and the infant was discharged on day four of life in good condition.

On day seven of life, he presented with vomiting, initially non-bilious and later bilious, accompanied by visible jaundice. On admission, his abdomen was distended and soft, with no organomegaly. He continued to pass urine and stools. He had lost 9.3% of his weight and weighed 2.71 kg on admission. Arterial blood gas analysis showed metabolic alkalosis, likely secondary to persistent vomiting. Serum electrolytes revealed hypochloremia and hypokalemia, consistent with gastric fluid loss.

**Figure 1:**
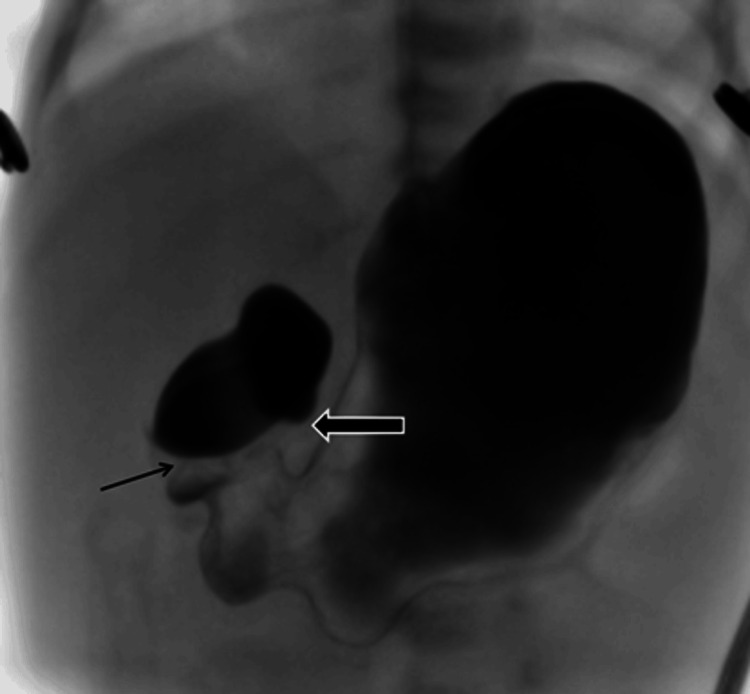
Intraoperative image showing preduodenal portal vein crossing anterior to the duodenum causing obstruction. AP projection of the upper GI examination shows contrast in the stomach and dilated proximal duodenum with an obstruction at the level of the second part of duodenum (solid arrow). The gastric pylorus is seen separately (thin arrow).

**Figure 2:**
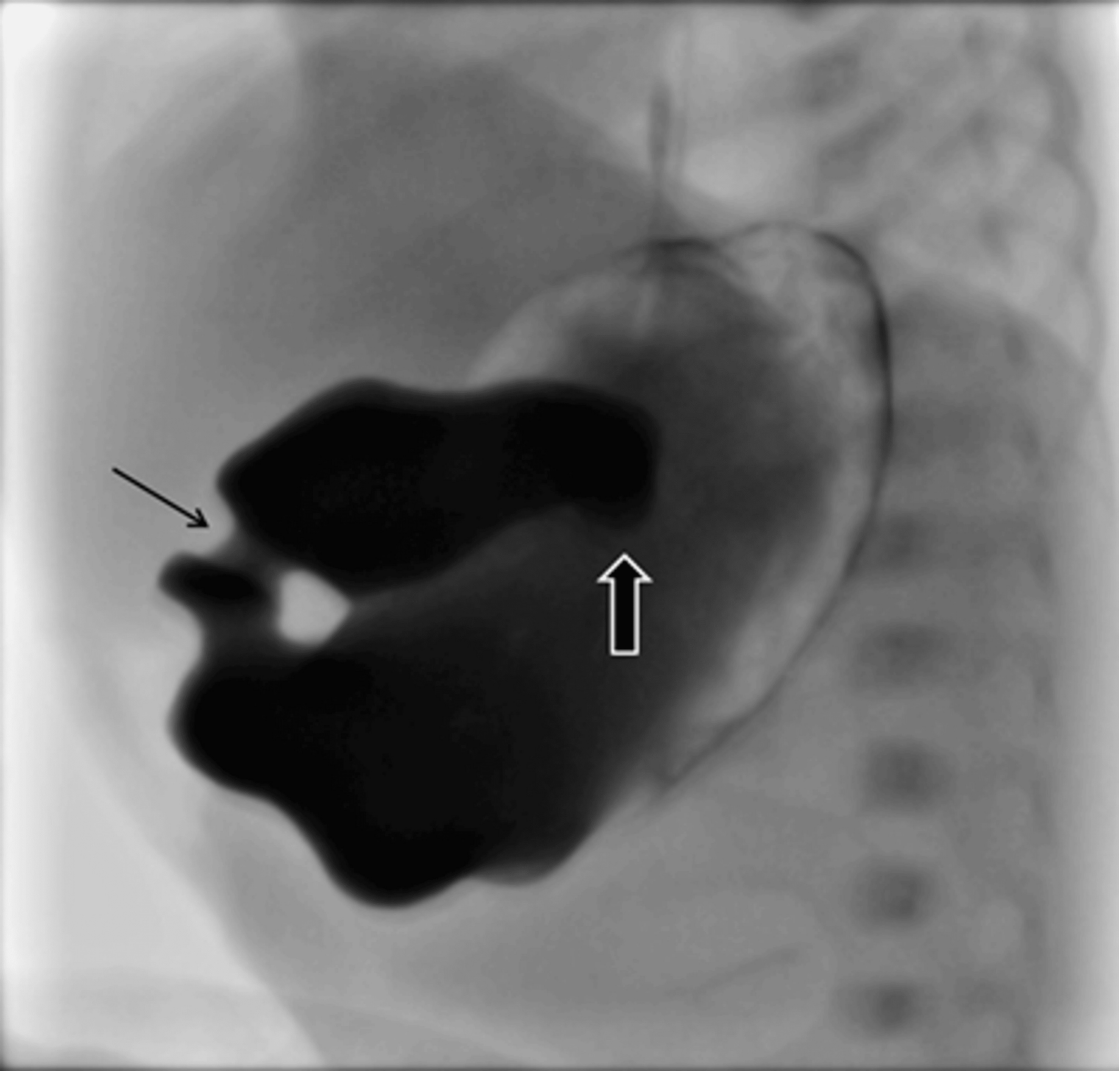
Intraoperative view demonstrating midgut non-rotation with abnormal positioning of bowel loops. Lateral projection of the upper GI examination shows contrast in the stomach and dilated proximal duodenum with an obstruction at the level of the second part of the duodenum (solid arrow). The gastric pylorus is seen separately (thin arrow).

An abdominal X-ray showed gastric distension with limited distal bowel gas, raising suspicion of proximal duodenal obstruction. These radiographic findings, although suggestive of proximal duodenal obstruction, are non-specific and require further imaging, as seen in the present case. Abdominal ultrasound excluded pyloric stenosis but revealed preduodenal portal vein (PDPV). A fluoroscopic contrast study demonstrated mechanical obstruction at the second part of the duodenum, consistent with extrinsic compression by a PDPV, with possible associated malrotation (Figures 1, 2).

**Figure 3:**
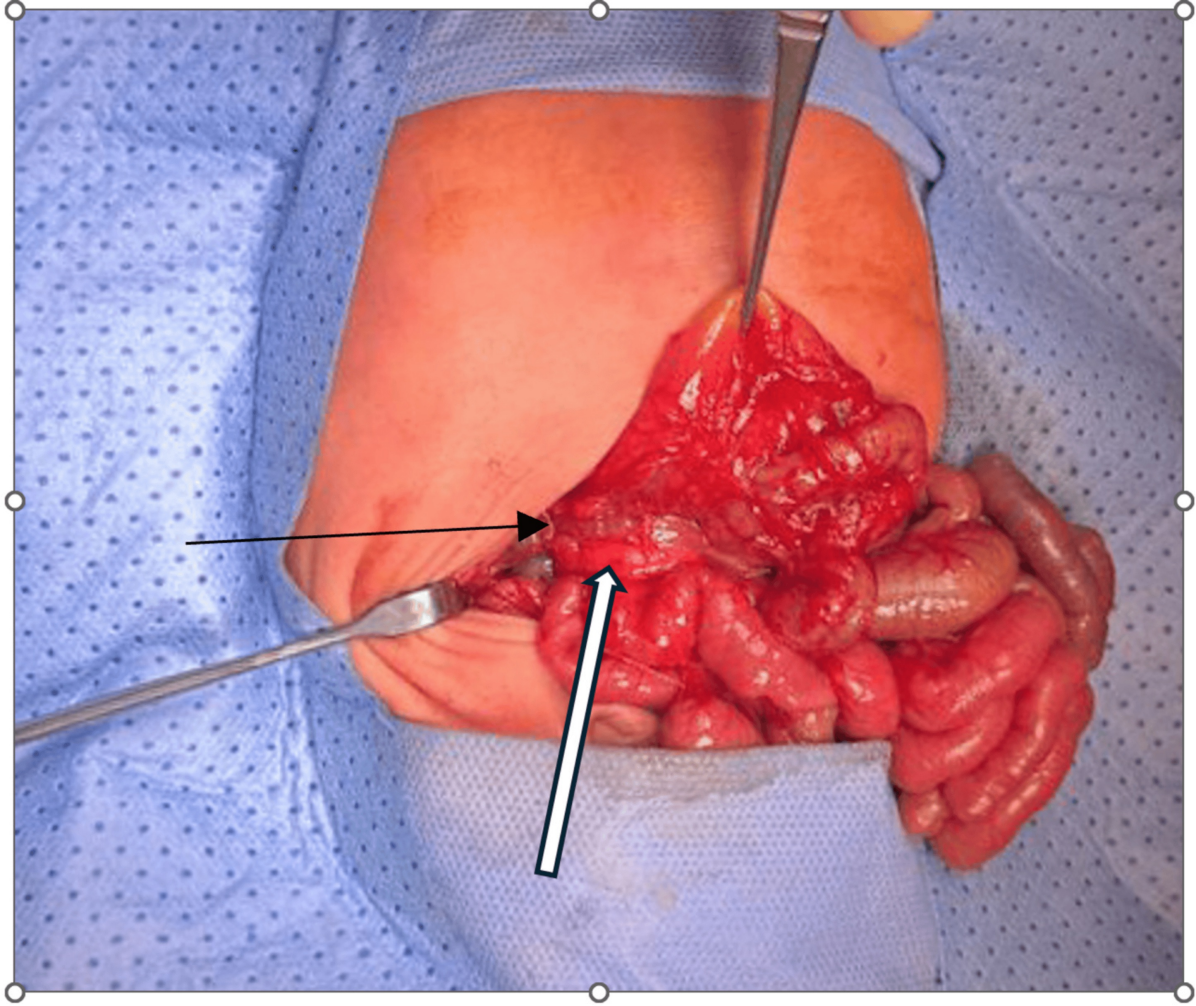
Duodenum (thick arrow) and portal vein (thin arrow).

On day nine of life, we proceeded with an exploratory laparotomy. Intraoperatively, a markedly dilated stomach and first portion of the duodenum were noted, consistent with proximal obstruction. Multiple Ladd’s bands were identified crossing the duodenum, and non-rotation of the midgut was confirmed. Most importantly, the portal vein was found coursing anterior to the duodenum, consistent with a preduodenal portal vein (PDPV) (Figure 3). After carefully releasing the Ladd’s bands, a side-to-side duodenoduodenostomy was performed between D1 and D3, taking care to preserve the anomalous portal vein. An appendectomy was also completed as part of Ladd's procedure. A peripherally inserted central catheter (PICC) was placed for parenteral nutrition.

Postoperatively, the infant required 24 h of mechanical ventilation. He was kept nil by mouth and maintained on total parenteral nutrition (TPN). Following a reassuring abdominal X-ray on day 14, feeds were reintroduced and gradually advanced; full oral demand feeding was achieved by day 20. The PICC line was removed the same day.

During the postoperative course, he developed anemia on day 13 without overt bleeding. Ultrasound suggested minor postoperative oozing; however, the original imaging could not be retrieved from the hospital archive due to a system data loss. He was transfused with packed red cells (20 mL/kg), resulting in hemoglobin stabilization.

On day 13, a new systolic murmur was detected. Echocardiography demonstrated a small muscular ventricular septal defect with peripheral pulmonary artery stenosis; the imaging could not be retrieved due to system data loss. The infant remained hemodynamically stable, and cardiology follow-up was arranged. By postoperative day 20, he was feeding well and gaining weight. Jaundice had resolved, inflammatory markers had normalized, and he was discharged home in stable condition. Over a two-month follow-up period, the child maintained appropriate growth and achieved expected developmental milestones for age, indicating satisfactory progress without any features of failure to thrive.

## Discussion

Preduodenal portal vein is an uncommon congenital anomaly characterized by anterior positioning of the portal vein relative to the duodenum, with potential to cause extrinsic duodenal obstruction. Although PDPV may remain clinically silent and be discovered incidentally in adulthood, it carries particular clinical significance in neonates, where it may present with signs of proximal intestinal obstruction [6].

The reported incidence of PDPV is low, estimated at approximately one in 10,000 live births, and it accounts for nearly 4% of cases of congenital duodenal obstruction [8]. In neonates, clinical presentation commonly includes bilious vomiting, abdominal distension, and feeding intolerance when obstruction is significant [8]. The condition frequently coexists with intestinal malrotation or non-rotation of the midgut. Laterality defects such as situs inversus and polysplenia have also been described [3,9]. These associations underscore the importance of comprehensive anatomical evaluation once PDPV is identified.

Radiologic evaluation plays a central role in diagnosis. Plain abdominal radiographs may demonstrate a classic double-bubble sign suggestive of proximal duodenal obstruction, although this finding is non-specific. Upper gastrointestinal contrast studies are more informative, delineating the level of obstruction and identifying associated malrotation if present. Cross-sectional imaging, such as computed tomography or magnetic resonance imaging, is seldom required in neonates but may assist in vascular mapping in older children or adults. While prenatal ultrasonography may raise suspicion for duodenal obstruction, PDPV itself is rarely identified antenatally [7,10].

Surgical intervention is indicated when PDPV results in symptomatic obstruction. The preferred operative management is duodenoduodenostomy, typically performed in a diamond-shaped or side-to-side fashion to bypass the obstruction caused by the anomalous vein [6]. Duodenojejunostomy may be considered when duodenal mobilization is limited. In cases associated with malrotation or non-rotation, a Ladd’s procedure with appendectomy should be performed concurrently to prevent future volvulus. Careful intraoperative identification of the portal vein is critical to avoid vascular injury.

Postoperative management usually involves a period of parenteral nutrition with gradual advancement to enteral feeding as bowel function returns. Reported outcomes are generally favorable, with most neonates achieving full oral intake within days to weeks following surgery [6]. Long-term prognosis largely depends on the presence and severity of associated congenital anomalies.

The present case reinforces the importance of early recognition, meticulous operative planning, and structured postoperative care, including temporary total parenteral nutrition and staged advancement of feeds. Reporting such cases contributes to expanding the existing literature and enhancing awareness of this rare but surgically significant anomaly.

## Conclusions

Preduodenal portal vein is a rare congenital vascular anomaly that should be considered in neonates presenting with bilious vomiting and proximal intestinal obstruction, particularly when associated anomalies such as intestinal malrotation are suspected. Although it may be asymptomatic, its presence has significant surgical implications due to the risk of inadvertent vascular injury. Early diagnosis through appropriate imaging, meticulous intraoperative identification, and tailored surgical management are essential to ensure favorable outcomes. This case highlights the importance of maintaining a high index of suspicion for rare anatomical variants and underscores the need for careful operative planning in neonatal duodenal obstruction.
